# Emergency Cholecystectomy in Patients Classified as High Risk According to the Tokyo Guidelines 2018: A Real‐World Analysis

**DOI:** 10.1002/ags3.70209

**Published:** 2026-03-02

**Authors:** Satoshi Mii, So Yamaki, Daisuke Hashimoto, Kazuki Matsumura, Hiroyuki Ishida, Hidetaka Miyazaki, Yuki Matsui, Yuji Ikeda, Tsukasa Ikeura, Sohei Satoi

**Affiliations:** ^1^ Department of Pancreatobiliary Surgery Kansai Medical University Hirakata Japan; ^2^ Third Department of Internal Medicine Kansai Medical University Hirakata Japan; ^3^ Division of Surgical Oncology University of Colorado Anschutz Medical Campus Aurora Colorado USA

**Keywords:** acute cholecystitis, emergency cholecystectomy, perioperative outcomes, risk stratification, Tokyo guideline 2018

## Abstract

**Aim:**

Emergency cholecystectomy for acute cholecystitis remains controversial in patients classified as high risk by the Tokyo Guidelines 2018 (TG18), although surgery is often unavoidable in real‐world emergency settings. The perioperative risk profile of this TG18 non‐recommended population remains insufficiently defined. The objective of this study was to examine perioperative outcomes in patients undergoing emergency cholecystectomy against TG18 recommendations, while also exploring clinical factors associated with actual operative risk.

**Methods:**

This retrospective cohort study included 252 consecutive patients who underwent emergency cholecystectomy for acute cholecystitis between 2018 and 2025. Patients were stratified into TG18 emergency‐surgery‐recommended and non‐recommended groups. Perioperative outcomes were compared, and independent risk factors of major postoperative complications, defined as Clavien‐Dindo grade≥III events, were evaluated.

**Results:**

Major postoperative complications occurred in 11.9% of patients and were significantly more frequent in the TG18 non‐recommended group than in the recommended group (18.0% vs. 2.9%, *p* < 0.001). In multivariable analysis, American Society of Anesthesiologists physical status classification ≥ 3 and preoperative shock status were independent predictors of major postoperative complications, whereas age and Charlson Comorbidity Index were not. Exploratory stratification of the non‐recommended cohort demonstrated substantial heterogeneity in risk, with comparatively low complication rates observed in patients without physiological instability.

**Conclusion:**

Emergency cholecystectomy may be feasible in carefully selected TG18 non‐recommended patients. Perioperative risk appears to be driven by physiological instability rather than chronological age or comorbidity burden, supporting a more individualized approach to surgical decision‐making.

## Graphical abstract

Emergency cholecystectomy was evaluated in patients with acute cholecystitis classified as non‐recommended for surgery by the Tokyo Guidelines 2018. Major postoperative complications, rather than mortality, better reflected operative risk. Physiological instability, particularly ASA‐PS ≥ 3 and shock status, identified high‐risk patients, suggesting that surgical decision‐making should prioritize real‐time physiological status over age or comorbidity burden.

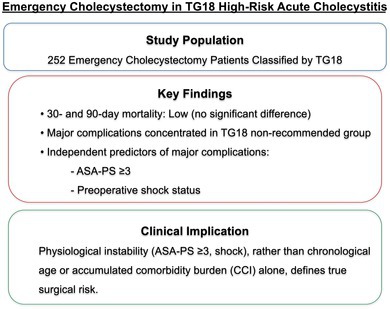

## 1 Introduction

Gallstones are the predominant etiology, affecting approximately 10%–15% of the general population, of whom 1%–4% annually develop gallstone‐related complications such as acute cholecystitis (AC) [1, 2, 3]. Large cohort studies from Japan and Taiwan have reported a 30‐day mortality of approximately 1.1% for AC [4]. The Tokyo Guidelines 2018 (TG18) [5, 6] provide criteria for diagnosis, severity grading, and treatment algorithms for AC, recommending early laparoscopic cholecystectomy for Grade I or II disease in patients with a Charlson Comorbidity Index (CCI) < 6. In contrast, TG18 advocates conservative or non‐surgical approaches, such as percutaneous transhepatic gallbladder drainage (PTGBD), in patients with Grade III disease, those with a high comorbidity burden (CCI ≥ 6), or an American Society of Anesthesiologists physical status classification (ASA‐PS) ≥ 3 [6, 7]. However, in real‐world emergency settings, surgery cannot be always avoided, particularly in patients with diffuse peritonitis, gallbladder perforation, or failure of drainage therapy. These patients often fall outside TG18‐recommended criteria yet still require operative intervention. Despite this clinical reality, the perioperative risk profile and outcomes of this TG18 non‐recommended population remain insufficiently characterized. This study aimed to evaluate perioperative outcomes in patients who underwent emergency cholecystectomy despite TG18 non‐recommendation and to identify clinical factors associated with true operative risk.

## 2 Methods

### 2.1 Study Design and Patient Selection

This retrospective cohort study included consecutive patients who underwent emergency cholecystectomy for acute cholecystitis at our institution between September 2018, and October 2025. Diagnosis and severity grading were determined according to the Tokyo Guideline 2018 (TG18) criteria. Patients were first classified into TG18 emergency‐surgery‐recommended and the non‐recommended groups based on guideline‐defined surgical indications, and perioperative characteristics and outcomes were compared between these two groups. Patients were also stratified by disease severity into Grade I/II versus Grade III acute cholecystitis according to the TG18 severity classification. This severity‐based comparison was performed because TG18 recommends early laparoscopic cholecystectomy for Grade I/II patients when their systemic condition permits, whereas a more cautions or individualized approach is generally considered for Grade III patients. In addition, a predefined subgroup analysis was conducted to identify high‐risk patients within the TG18 non‐recommended group.

### 2.2 Definition of TG18 Non‐Recommended Classification

TG18 non‐recommended status for emergency cholecystectomy was operationally defined based on comorbidity burden, specifically an ASA‐PS ≥ 3 and/or a CCI ≥ 6. Although Grade III acute cholecystitis represents advanced disease severity, TG18 allows early surgery in selected Grade III patients with reversible organ dysfunction and favorable response to initial treatment. Therefore, Grade III severity alone was not used to define non‐recommended status and was analyzed separately as a severity descriptor.

### 2.3 Definition of Preoperative Shock Status

Shock status was evaluated preoperatively at the time of surgical decision‐making and defined according to TG18 criteria as the requirement for dopamine ≥ 5 μg/kg/min and/or norepinephrine administration.

### 2.4 Data Collection and Clinical Variables

Baseline demographic and clinical information, including age, sex, body mass index, comorbidities, and laboratory findings at presentation, was extracted from electronic medical records. Physiological status was assessed using the ASA‐PS [8], and comorbidity burden was evaluated using the age‐adjusted CCI [4].

### 2.5 Surgical Management and Operative Strategy

Although TG18 generally discourages emergency cholecystectomy in high‐risk patients (ASA‐PS ≥ 3 and/or CCI ≥ 6), emergency cholecystectomy was performed in selected patients when emergency gallbladder drainage was considered difficult or unsafe. Indications included suspected gallbladder perforation, gangrenous cholecystitis, presence of ascites, anticoagulation therapy, or inadequate control of inflammation or pain despite conservative management, following multidisciplinary discussion with gastroenterologists.

Laparoscopic cholecystectomy was the standard operative approach whenever feasible. Open surgery was selected when extensive adhesions were anticipated due to prior laparotomies or when the patient's systemic condition was severely compromised. Conversion from laparoscopic to open surgery was performed intraoperatively in cases of uncontrolled bleeding, severe inflammation, or difficulty in achieving secure anatomical identification.

### 2.6 Definition of Bailout Surgery

When the critical view of safety could not be achieved, bailout strategies were adopted to minimize the risk of bile duct injury. “Bailout surgery” was defined as any intentional deviation from standard laparoscopic cholecystectomy undertaken for safety reasons, consistent with the concept described by Strasberg et al. [9]. Bailout procedures included: (1) conversion to open cholecystectomy; (2) subtotal cholecystectomy with partial wall preservation and cystic duct control; (3) closure‐type subtotal resection using ligation or stapling; (4) omental plugging of the gallbladder stump [10]; and (5) coverage of an open gallbladder stump using omentum or round ligament of the liver in the absence of bile leakage. For classification purposes, when multiple bailout techniques were used in a single case, patients were categorized according to the primary operative strategy.

### 2.7 Management of Concomitant CBD Stones and Intraoperative Damage‐Control

For patients with concomitant common bile duct (CBD) stones, endoscopic management was generally performed before cholecystectomy. However, when abdominal pain or inflammatory findings persisted despite endoscopic clearance of CBD stones was incomplete, surgical management of CBD stone was performed concurrently with cholecystectomy. In rare cases where hemorrhage could not be adequately controlled, packing and open abdominal management were used as a damage‐control strategy.

### 2.8 Postoperative Management

Postoperative intensive care unit (ICU) admission was provided when clinically indicated, particularly for patients exhibiting physiologic instability, high operative burden, or shock at presentation. Surgeons experienced in emergency hepatobiliary and pancreatic surgery participated in all high‐risk procedures.

### 2.9 Endpoints

The primary endpoint was the occurrence of major postoperative morbidity during the index hospitalization, defined as Clavien–Dindo [11] grade III–V complications. These events represent clinically meaningful adverse outcomes requiring surgical, endoscopic, or radiological intervention or resulting in life‐threatening organ dysfunction. In‐hospital mortality was included with this classification as the most severe complication. Major complications were subclassified into (1) surgical complications, defined as adverse events directly related to the surgical procedure (e.g., bile leakage, intra‐abdominal abscess requiring intervention, postoperative bleeding), and (2) medical complications, defined as events predominantly attributable to the patient's underlying physiological status or comorbidities rather than the procedure itself (e.g., respiratory failure, cardiac events, acute kidney injury). Secondary endpoints included operative outcomes, postoperative ICU admission, ICU length of stay, unplanned reoperation or reintervention, postoperative hospital length of stay, and 30‐ and 90‐day mortality. ICU length of stay was calculated as the total number of calendar days spent in the ICU, with patients not requiring ICU care recorded as 0 days.

### 2.10 Statistical Analyses

Statistical analyses were performed using EZR (Saitama Medical Center, Jichi Medical University, Saitama, Japan), which is a graphical user interface for R [12]. Continuous variables with non‐normal distributions were summarized as medians with interquartile ranges, whereas categorical variables were presented as frequencies and percentages. For univariable comparisons between two groups, continuous variables were compared using the Mann–Whitney *U* test, as most variables were not normally distributed. Categorical variables were compared using the chi‐square test or Fisher's exact test, as appropriate. To identify potential risk factors for major postoperative complications, univariable analyses were performed with preoperative patient‐related variables entered as explanatory variables. Candidate variables for multivariable analysis were selected using a stepwise procedure, with variables showing a *p* < 0.10 in univariable analysis considered for inclusion. Multicollinearity was evaluated using variance inflation factors (VIF). A VIF < 5 was considered acceptable, and variables with VIF > 10 were considered to have significant collinearity. Although the VIF for AC Grade III was moderately elevated, the variable was retained in the multivariable model because of its strong clinical relevance as a key determinant of disease severity according to TG18. Multivariable logistic regression analysis was then performed to identify independent predictors of the primary endpoint. A *p* < 0.05 was considered statistically significant.

## 3 Results

### 3.1 Patient Characteristics

**FIGURE 1:**
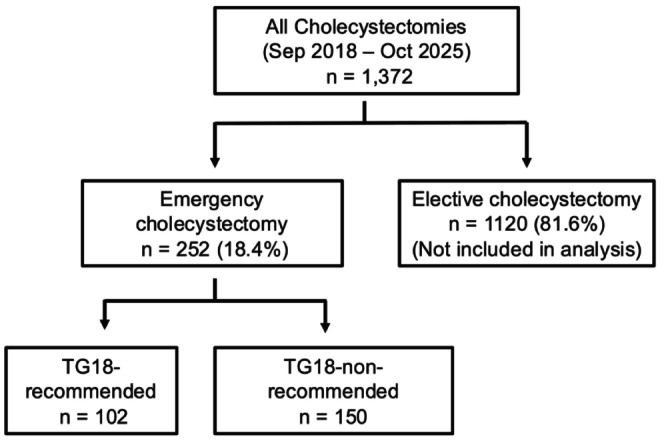
Institutional volume of cholecystectomy and study population flow. (A) Annual volume of cholecystectomy performed at our institution between September 2018 and October 2025. (B) Flowchart showing patient selection for the present study. Among 1372 cholecystectomies performed during the study period, 252 patients (18.4%) underwent emergency cholecystectomy for acute cholecystitis and were evaluated according to the Tokyo Guidelines 2018 (TG18) treatment algorithm. TG18, Tokyo Guidelines 2018.

During the study period from September 2018 to October 2025, a total of 1372 cholecystectomies were performed at our institution. Among these, 252 patients (18.4%) underwent emergency cholecystectomy for acute cholecystitis and constituted the study cohort. According to TG18 criteria, 102 patients were classified into the emergency‐surgery‐recommended group and 150 into the non‐recommended group (Figure 1). Baseline patient characteristics are summarized in Table 1. The median age of the entire cohort was 74 years, and 22.6% of patients presented with Grade III AC. Nearly one‐third of patients had ASA‐PS ≥ 3, and the median CCI was 6. Importantly, elderly patients were substantially represented, with 29.4% of the cohort aged ≥ 80 years. Preoperative shock status was present in 9 patients (3.6%), all of whom were included in the TG18 non‐recommended group.

**TABLE 1:** Baseline characteristics of the patients and comparison of outcomes between TG18 emergency‐surgery‐recommended and non‐recommended group.

Variable	Overall (*n* = 252)	TG18‐recommended (*n* = 102)	TG18‐non‐recommended (*n* = 150)	*p*
*Preoperative factors*				
Age, median (IQR)	74.0 [65.8, 81.0]	66.00 [54.00, 75.00]	77.00 [72.00, 83.00]	< 0.001
Sex, male, *n* (%)	161 (63.9)	62 (60.8)	99 (66.0)	0.424
BMI, median (IQR)	23.1 [21.0, 25.7]	24.26 [22.41, 26.98]	22.17 [20.41, 24.54]	< 0.001
ASA–PS, *n* (%)				< 0.001
1	23 (9.1)	22 (21.6)	1 (0.7)	
2	149 (59.1)	80 (78.4)	69 (46.0)	
3	78 (31.0)	0 (0.0)	78 (52.0)	
4	2 (0.8)	0 (0.0)	2 (1.3)	
Age‐adjusted CCI, median (IQR)	6 [4, 7]	4.00 [3.00, 5.00]	7.00 [6.00, 8.00]	< 0.001
WBC count, ×10^3^/μL, median (IQR)	11.2 [8.0, 15.1]	11.3 [8.1, 15.6]	11.1 [7.9, 14.8]	0.545
CRP (mg/dL), median (IQR)	15.5 [6.0, 22.5]	13.54 [2.22, 20.83]	16.46 [9.10, 23.54]	0.002
Platelet count, ×10^3^/μL, median (IQR)	19.4 [14.3, 26.0]	21.60 [16.55, 28.45]	18.10 [12.67, 24.98]	< 0.001
PT (%), median (IQR)	77.0 [65.9, 88.2]	83.30 [71.50, 91.30]	74.35 [61.58, 84.40]	< 0.001
Albumin (g/dL), median (IQR)	3.2 [2.6, 3.7]	3.60 [3.10, 4.15]	2.90 [2.40, 3.35]	< 0.001
Total bilirubin (mg/dL), median (IQR)	1.0 [0.7, 1.8]	1.05 [0.70, 1.60]	1.00 [0.60, 1.80]	0.413
Creatinine (mg/dL), median (IQR)	0.88 [0.67, 1.21]	0.83 [0.65, 1.04]	0.94 [0.70, 1.55]	0.006
Acute Cholecystitis Grade, *n* (%)				< 0.001
Grade I	25 (9.9)	12 (11.8)	13 (8.7)	
Grade II	170 (67.5)	82 (80.4)	88 (58.7)	
Grade III	57 (22.6)	8 (7.8)	49 (32.7)	
Use of anticoagulant or antiplatelet agents, *n* (%)	73 (29.0)			0.001
ACT	27 (10.7)	6 (5.9)	21 (14.0)	
APT	33 (13.1)	8 (7.8)	25 (16.7)	
DATT	2 (0.8)	0 (0.0)	2 (1.3)	
DAPT	11 (4.4)	1 (1.0)	10 (6.7)	
Onset‐to‐surgery interval (days), median (IQR)	3.00 [2.00, 4.00]	3.00 [2.00, 4.00]	3.00 [2.00, 4.00]	0.762
*Intraoperative findings*				
Operation time (min), median (IQR)	125.00 [91.00, 164.25]	121.00 [91.25, 163.00]	127.00 [91.00, 165.50]	0.573
Intraoperative bleeding (mL), median (IQR)	20.00 [0.00, 150.00]	5.00 [0.00, 65.00]	50.00 [0.00, 218.75]	0.002
Intraoperative blood infusion, *n* (%)	22 (8.7)	1 (1.0)	21 (14.0)	< 0.001
Surgical approach, *n* (%)				0.004
Laparoscopic surgery	195 (77.4)	88 (86.3)	107 (71.3)	
Conversion to open surgery	38 (15.1)	12 (11.8)	26 (17.3)	
Open surgery	19 (8.4)	2 (2.0)	17 (11.3)	
Surgical procedure, *n* (%)				0.01
Laparoscopic cholecystectomy	182 (72.2)	87 (85.3)	95 (63.3)	
Cholecystectomy	58 (23.0)	12 (11.8)	46 (30.7)	
Cholecystectomy with CBD stones extraction	5 (2.0)	1 (1.0)	4 (2.7)	
Laparoscopic cholecystectomy with CBD stones extraction	3 (1.2)	1 (1.0)	2 (1.3)	
Cholecystectomy and OAM	2 (0.8)	0 (0.0)	2 (1.3)	
Others	2 (0.8)	1 (1.0)	1 (0.7)	
Bailout surgery, *n* (%)	64 (25.4)	23 (22.5)	41 (27.3)	0.46
Intraoperative bile duct injury, *n* (%)	2 (0.8)	1 (1.0)	1 (0.7)	1
*Postoperative outcomes*				
Postoperative bile leakage, *n* (%)	6 (2.4)	2 (2.0)	4 (2.7)	1
Re‐operation, *n* (%)	5 (2.0)	0 (0.0)	5 (3.3)	0.08
Postoperative complications of Clavien–Dindo grade≥III, *n* (%)	30 (11.9)	3 (2.9)	27 (18.0)	< 0.001
Detail of postoperative complications				0.001
Surgical complications (procedure‐related)	16 (6.3)	2 (2.0)	14 (9.3)	
Medical complications (patient condition‐related)	14 (5.6)	1 (1.0)	13 (8.7)	
ICU admission, *n* (%)	80 (31.7)	8 (7.8)	72 (48.0)	< 0.001
Length of ICU stay, days, median (IQR)	0 [0, 15]	0.0 (0.0, 0.0)	0.0 [0.0, 2.0]	< 0.001
Gangrenous cholecystitis on pathology, *n* (%)	99 (39.3)	28 (27.5)	71 (47.3)	0.002
Length of postoperative hospital stay (days), median (IQR)	8.00 [2, 167]	6.0 [4.3, 9.0]	10.0 [7.0, 20.0]	< 0.001
30‐day mortality, *n* (%)	3 (1.2)	0 (0.0)	3 (2.0)	0.27
90‐day mortality, *n* (%)	4 (1.6)	0 (0.0)	4 (2.7)	0.15

*Note:* TG18 emergency surgery‐recommended and ‐non‐recommended groups were defined according to the TG18 criteria. “Others” included laparoscopic cholecystectomy with gastrostomy (*n* = 1) and diagnostic laparoscopy alone due to extensive pericholecystic involvement from metastatic pancreatic cancer (*n* = 1). Among patients who developed major postoperative complications (Clavien‐Dindo grade III–V), complications were further subclassified for descriptive purpose into surgical (procedure‐related) and medical (patient condition‐related) categories based on the primary cause of event.

Abbreviations: APT, antiplatelet therapy; ASA‐PS, American Society of Anesthesiologists physical status classification; BMI, body mass index; CBD stones, common bile duct stones; CCI, Charlson Comorbidity Index; CRP, C related protein; DAPT, dual antiplatelet therapy; DATT, dual antithrombotic therapy; ICU, intensive care unit; IQR, interquartile range; OAM, open abdominal management; PT, prothrombin time; anticoagulant therapy; TG18, Tokyo Guidelines 2018; WBC, white blood cell.

### 3.2 Patient Classification

Among the study population, 80 patients met the ASA‐PS ≥ 3 criterion, 132 met the CCI ≥ 6 criterion, and 62 met both criteria. Grade III acute cholecystitis was observed in 57 patients, of whom 49 were classified as TG18 non‐recommended based on comorbidity criteria and 8 were classified as TG18 recommended because their organ dysfunction was reversible and their clinical condition improved with initial treatment. The distribution and overlap of these comorbidity‐based criteria are shown in Table S1.

### 3.3 Operative Findings and Procedures

Laparoscopic surgery was attempted in 195 patients (77.4%). Conversion to open surgery occurring in 38 patients (15.1%), and open cholecystectomy was performed initially in 19 patients (8.4%). Procedure‐related adverse events were uncommon. Intraoperative bile duct injury occurred in 2 patients (0.8%), and no major vascular injury was observed. Transfusion‐requiring bleeding occurred in 22 patients (8.7%), predominantly in the TG18 non‐recommended group. Five patients (2.0%) required reoperation or reintervention. The median operative time was 125 min, and median intraoperative bleeding was 20 mL (IQR, 0–150 mL). Intraoperative blood transfusion was required in 8.7% of cases, predominantly in the TG18 non‐recommended group. Intraoperative bile duct injury was rare, occurring in only 2 patients (0.8%). Bailout surgery was performed in 64 patients (25.4%). Among these cases, various types of subtotal cholecystectomy and related bailout techniques were employed, as detailed in Table S2. Gangrenous cholecystitis was confirmed pathologically in 99 patients (39.3%).

### 3.4 Postoperative Outcomes

Major postoperative complications (Clavien–Dindo grade≥III) occurred in 30 patients (11.9%), including 16 (6.3%) surgical (procedure‐related) complications and 14 (5.6%) medical (systemic or cardiopulmonary) complications. Detailed classifications and corresponding interventions are provided in Table S3.

ICU admission was required in 80 patients (31.7%). The median ICU length of stay was 0 days (IQR, 0–15), and the median postoperative length of hospital stay was 8 days (IQR, 2–167). Five patients (2.0%) required unplanned reintervention. Two patients underwent open abdominal management with gauze packing for uncontrolled intraoperative bleeding during open cholecystectomy and required relaparotomy on the following day. One patient underwent exploratory laparotomy on postoperative day (POD) 11 after laparoscopic cholecystectomy for suspected gastrointestinal perforation; however, no perforation was identified, and the procedure was limited to exploration. Another patient underwent exploratory laparotomy on POD 14 after open cholecystectomy for suspected bowel necrosis, which was not confirmed intraoperatively. One additional patient required emergency laparotomy on POD 7 following open cholecystectomy due to intraperitoneal perforation of an endoscopic nasobiliary drainage tube placed during endoscopic bile duct stone extraction, and the tube position was subsequently corrected. Perioperative mortality was low: 30‐day and 90‐day mortality rates were 1.2% and 1.6%, respectively. Details of these deaths are described below. Three patients (1.2%) died within 30 days. One patient who underwent laparoscopic cholecystectomy with common bile duct stone extraction died of sepsis secondary to a liver‐bed abscess on POD 11. Another patient who underwent open cholecystectomy developed postoperative cholangitis and respiratory failure, both of which improved, but subsequently died of acute heart failure on POD 28. The third 30‐day death occurred in a patient receiving treatment for maxillary sinus cancer; aspiration pneumonia requiring nasal high‐flow oxygen therapy and candidemia resolved, but the patient died of non‐occlusive mesenteric ischemia on POD 20. One additional patient (0.4%) died within 90 days; this patient had Child–Pugh class C cirrhosis, developed intra‐abdominal bleeding and sepsis after open cholecystectomy, and ultimately died of liver failure on POD 60.

### 3.5 Comparison of Outcomes Between TG18 Emergency‐Surgery‐Recommended and Non‐Recommended Group

Perioperative outcomes stratified by TG18 recommendation status are summarized in Table 1.

Major postoperative complications were significantly more frequent in the non‐recommended group than in the recommended group (18.0% vs. 2.9%, *p* < 0.001), affecting both surgical (9.3% vs. 2.0%) and medical complications (8.7% vs. 1.0%). Secondary perioperative outcomes demonstrated substantial differences between groups. Patients in the TG18 non‐recommended group were older and exhibited poorer physiological status, with higher ASA‐PS and CCI values. They are also more frequently presented with Grade III AC. Systemic inflammation and organ dysfunction on laboratory evaluation were more severe in this cohort. Although operative time was comparable, intraoperative bleeding and blood transfusion requirements were both markedly higher in the non‐recommended group (50 mL vs. 5 mL; transfusion 14.0% vs. 1.0%). Laparoscopic surgery was less frequently performed, and conversion to open surgery was more common (17.3% vs. 11.8%). ICU admission occurred significantly more often (48.0% vs. 7.8%, *p* < 0.001). Although the median ICU stay was 0 days in both groups, the IQR was wider in the TG18 non‐recommended group (0–2 vs. 0–0), suggesting a subset of patients requiring prolonged ICU management. The postoperative hospital stay was notably longer (10 vs. 6 days, *p* < 0.001). Clinically meaningful adverse events were thus predominantly concentrated in the TG18 non‐recommended group.

### 3.6 Outcomes According to the Severity of Acute Cholecystitis

Comparisons according to disease severity are presented in Table 2.

**TABLE 2:** Outcomes according to the severity of acute cholecystitis.

Variable	Acute cholecystitis grade		*p*
	Grade I/II (*n* = 195)	Grade III (*n* = 57)	
*Preoperative factors*			
Age, median (IQR)	74.0 [64.0, 81.0]	75.0 [71.0, 81.0]	0.16
Gender, male, *n* (%)	119 (61.0)	42 (73.7)	0.09
BMI, median (IQR)	23.3 [21.2, 25.7]	22.9 [20.4, 25.6]	0.23
ASA–PS ≥ 3, *n* (%)	44 (22.6)	36 (63.2)	< 0.001
Age‐adjusted CCI ≥ 6, *n* (%)	93 (47.7)	40 (70.2)	0.003
WBC count, ×10^3^/μL, median (IQR)	10.9 [8.0, 15.2]	11.3 [8.3, 14.7]	0.88
CRP (mg/dL), median (IQR)	13.7 [4.7, 21.9]	19.1 [12.2, 25.9]	0.003
Platelet count, ×10^3^/μL, median (IQR)	20.7 [15.9, 26.4]	14.4 [7.7, 23.5]	< 0.001
PT (%), median (IQR)	80.3 [70.2, 89.9]	63.1 [50.4, 79.9]	< 0.001
Albumin (g/dL), median (IQR)	3.3 [2.7, 3.8]	2.8 [2.2, 3.3]	< 0.001
Total bilirubin (mg/dL), median (IQR)	1.0 [0.7, 1.7]	0.9 [0.6, 1.8]	0.93
Creatinine (mg/dL), median (IQR)	0.82 [0.64, 1.10]	1.39 [0.77, 2.33]	< 0.001
High intensity anti‐thrombotic therapy	25 (12.8)	15 (26.3)	0.02
*Intraoperative findings*			
Operation time (min), median (IQR)	126.0 [89.0, 160.5]	121.0 [105.0, 169.0]	0.54
Intraoperative bleeding (mL), median (IQR)	8.0 [0.0, 108.0]	104.0 [3.0, 400.0]	< 0.001
Intraoperative blood infusion, *n* (%)	9 (4.6)	13 (22.8)	< 0.001
Surgical approach, *n* (%)			0.03
Laparoscopic surgery	158 (81.0)	37 (64.9)	
Conversion to open surgery	26 (13.3)	12 (21.1)	
Open surgery	11 (5.6)	8 (14.0)	
Surgical procedure (%)			0.02
Laparoscopic cholecystectomy	148 (75.9)	34 (59.6)	
Cholecystectomy	38 (19.5)	20 (35.1)	
Cholecystectomy with CBD stones extraction	4 (2.1)	1 (1.8)	
Laparoscopic cholecystectomy with CBD stones extraction	3 (1.5)	0 (0.0)	
Cholecystectomy and OAM	0 (0.0)	2 (3.5)	
Others	2 (1.0)	0 (0.0)	
Bailout surgery, *n* (%)	46 (23.6)	18 (31.6)	0.23
Intraoperative bile duct injury, *n* (%)	1 (0.5)	1 (1.8)	0.40
*Postoperative outcomes*			
Postoperative bile leakage, *n* (%)	5 (2.6)	1 (1.8)	1.00
Re‐operation, *n* (%)	2 (1.1)	3 (5.5)	0.08
ICU admission, *n* (%)	41 (21.0)	39 (68.4)	< 0.001
Length of ICU stay, days, median (IQR)	0.0 (0.0, 0.0)	2.0 [0.0, 2.0]	< 0.001
Postoperative complications of Clavien–Dindo grade≥III, *n* (%)	19 (9.7)	11 (19.3)	0.06
Detail of postoperative complications			0.13
Surgical complications (procedure‐related)	10 (5.1)	6 (10.5)	
Medical complications (patient condition‐related)	9 (4.6)	5 (8.8)	
Length of postoperative hospital stay, days, median (IQR)	7.0 [5.0, 11.0]	13.0 [9.0, 29.0]	< 0.001
30‐day mortality, *n* (%)	3 (1.5)	0 (0.0)	1.00
90‐day mortality, *n* (%)	3 (1.6)	1 (1.8)	1.00

*Note:* “Others” included laparoscopic cholecystectomy with gastrostomy (*n* = 1) and diagnostic laparoscopy alone due to extensive pericholecystic involvement from metastatic pancreatic cancer (*n* = 1). Among patients who developed major postoperative complications (Clavien‐Dindo grade III–V), complications were further subclassified for descriptive purpose into surgical (procedure‐related) and medical (patient condition‐related) categories based on the primary cause of event.

Abbreviations: ACT, anticoagulant therapy; APT, antiplatelet therapy; ASA‐PS, American Society of Anesthesiologists physical status classification; BMI, body mass index; CBD stones, common bile duct stones; CCI, Charlson Comorbidity Index; CRP, C related protein; DAPT, dual antiplatelet therapy; DATT, dual antithrombotic therapy; ICU, intensive care unit; IQR, interquartile range; OAM, open abdominal management; PT, prothrombin time; TG 18, Tokyo Guidelines 2018; WBC, white blood cell.

Patients with Grade III disease demonstrated worse preoperative physiological and laboratory profiles. Intraoperative blood loss and transfusion requirements were significantly higher than those in Grade I/II disease. Open or conversion procedures were more frequently required in Grade III cases. Major postoperative complications occurred more frequently in Grade III disease, although statistical significance was not reached (19.3% vs. 9.7%, *p* = 0.06). ICU admission rates and postoperative hospital stay were significantly greater in this group.

### 3.7 Risk Factors for Major Postoperative Complications in the TG18 Non‐Recommended Group

Risk factor analysis for major postoperative complications (Clavien‐Dindo grade≥III) in the TG18 non‐recommended group is summarized in Table 3. In univariate analysis, several preoperative factors demonstrated significant associations with major complications, including ASA‐PS ≥ 3 and shock status. In the multivariate logistic regression model, ASA‐PS ≥ 3 (odds ratio [OR] 3.31; 95% confidence interval [CI] 1.11–9.90; *p* = 0.032) and shock status at presentation (OR 6.54; 95% CI 1.38–31.0; *p* = 0.018) remained independently associated with the development of major postoperative complications. Other factors, such as Grade III disease, CCI ≥ 6, and organ dysfunction variables, were not statistically significant in the adjusted model.

**TABLE 3:** Risk factors for major postoperative complications in the TG18 non‐recommended group.

	Univariate analysis			Multivariate analysis		
	Odds ratio	95% CI	*p*	Odds ratio	95% CI	*p*
Age ≥ 75 years	2.32	0.81–6.67	0.12			
ASA–PS ≥ 3	4.33	1.51–2.4	0.006	3.31	1.11–9.90	0.032
Age‐adjusted CCI ≥ 6	0.96	0.20–4.72	0.96			
Acute cholecystitis Grade III	1.7	0.69–4.24	0.25			
Shock status	10.7	2.36–48.9	0.002	6.54	1.38–31.0	0.018
Neurological dysfunction	1.76	0.18–17.7	0.63			
Respiratory dysfunction	1.52	0.30–7.85	0.61			
Renal dysfunction	1.47	0.38–5.75	0.58			
Coagulation disorder	2.26	0.72–7.13	0.16			
Hepatic dysfunction	1.32	0.26–6.67	0.74			
High intensity anti‐thrombotic therapy	0.72	0.23–2.29	0.58			

*Note:* Candidate variables for univariable logistic regression analyses were selected to represent preoperative patient condition, including age, American Society of Anesthesiologist Physical Status (ASA‐PS), Charlson Comorbidity Index (CCI), high‐intensity antithrombotic therapy, acute cholecystitis Grade III, and individual components defining Grade III severity. Variables with a *p* < 0.1 in univariable analyses were entered into the multivariable logistic regression model using a stepwise selection method. Shock status was defined as the requirement for dopamine ≥ 5 μg/kg/min and/or norepinephrine administration. Respiratory dysfunction was defined as a PaO2/FiO2 ratio < 300. Renal dysfunction was defined as oliguria or a serum creatinine level > 2.0 mg/dL. Coagulation disorder was defined as a platelet count < 100 000/μL. Hepatic dysfunction was defined as a prothrombin time‐international normalized ratio (PT‐INR) > 1.5.

### 3.8 Exploratory Risk Stratification Within the TG18 Non‐Recommended Cohort

Based on the identified risk factors, an exploratory stratified analysis was performed to evaluate whether lower‐risk subgroups could be identified among TG18 non‐recommended patients. Patients without ASA‐PS ≥ 3 and without shock (*n* = 70) showed relatively low rates of major postoperative complications (8.6%) and 90‐day mortality (1.4%). In contrast, those with ASA‐PS ≥ 3 only (*n* = 71) demonstrated higher complication rates (21.1%) and mortality (4.3%), while patients with shock (± ASA‐PS ≥ 3; *n* = 9) exhibited the highest complication rate (66.7%). Detailed results are provided in Table S4.

## 4 Discussion

This real‐world cohort study challenges the assumption that Tokyo Guidelines 2018 (TG18) non‐recommendation for emergency cholecystectomy uniformly indicates unacceptable surgical risk. The management of acute cholecystitis (AC) in surgically high‐risk patients remains one of the most challenging issues in emergency hepatobiliary surgery [13, 14, 15]. Although the TG18 provide structured recommendations for disease severity assessment and treatment selection [5, 6], real‐world clinical practice frequently encounters patients classified as “non‐recommended” who nevertheless require operative intervention because of disease progression, sepsis, or failure of conservative management. In this context, the present study provides contemporary real‐world data on perioperative outcomes and offers several important insights into risk stratification in this clinical dilemma. Importantly, the primary endpoint of major postoperative morbidity was significantly more frequent in the TG18 non‐recommended group, whereas overall mortality remained low. These findings indicate that complication‐based outcomes may more accurately reflect clinically meaningful surgical risk than mortality alone in this setting, where death is uncommon.

First, our findings demonstrate that the TG18 non‐recommended group is not a homogeneous population. Despite guideline‐based classification, perioperative outcomes varied substantially according to physiological reserve. Although this group as a whole exhibited worse perioperative outcomes than the recommended group, major postoperative complications were not uniformly observed. Instead, our results indicate that a subset of patients within the non‐recommended category can safely undergo emergency cholecystectomy, provided that severe physiological instability is absent. This observation suggests that the TG18 non‐recommendation group should not be interpreted as an absolute contraindication to surgery, but rather as a signal for heightened caution and individualized decision‐making.

This concept is consistent with those of previous reports addressing high‐risk AC [16]. Endo et al. emphasized that patients who underwent percutaneous cholecystostomy without subsequent cholecystectomy, often characterized by frailty, poor performance status, and advanced physiological deterioration, experienced the worst outcomes, whereas selected high‐risk patients who ultimately underwent definitive surgery achieved more favorable survival [16]. Similarly, the CHOCOLATE trial demonstrated that, even in patients defined as high risk by elevated APACHE II scores [17], laparoscopic cholecystectomy resulted in fewer major complications and lower healthcare costs than did percutaneous drainage alone [18]. Collectively, these data suggest that operative tolerance, rather than high‐risk designation itself, is the key determinant of postoperative outcome.

Additionally, an important message of our study is that chronological age alone should not define surgical risk. Although advanced age is frequently incorporated into guideline‐based risk stratification, age was not independently associated with major postoperative complications in our multivariable analysis. In contrast, ASA‐PS ≥ 3 and shock status were strong independent predictors of adverse outcomes. To further explore the clinical applicability of these findings, we conducted an exploratory stratified analysis within the TG18 non‐recommendation cohort based on combinations of ASA‐PS and shock status. This analysis demonstrated substantial heterogeneity in operative risk, with comparatively low complication rates observed among patients without physiological instability, whereas patients with ASA‐PS ≥ 3 and especially those presenting with shock exhibited markedly higher complication rates. However, these results should be interpreted as hypothesis‐generating rather than prescriptive, and prospective or external validation is required before such criteria can be incorporated into clinical decision‐making algorithms. Collectively, these findings imply that real‐time physiological instability, rather than accumulated comorbidity burden or chronological age, plays a dominant role in determining perioperative risk in emergency cholecystectomy. Consistent with these findings, a recent study validating TG18 [19] application in super‐elderly patients (≥ 85 years) demonstrated acceptable postoperative outcomes when physiological reserve was preserved, suggesting that chronological age alone should not define surgical eligibility. This interpretation is further supported by the age profile of our cohort, in which the median age was 74 years and nearly one‐third (29.4%) of patients were ≥ 80 years, indicating that advanced age alone did not translate into heightened postoperative risk. Accordingly, this emphasis on physiological status rather than comorbidity‐based indices may also explain why the CCI did not emerge as an independent predictor in our analysis. Notably, the CCI has primarily been used to predict mortality [4, 20, 21], rather than short‐term postoperative morbidity, which may limit its ability to discriminate acute surgical risk compared with ASA‐PS in emergency settings. This concept is supported by previous literature, in which ASA‐PS ≥ 3, rather than age alone was associated with increased morbidity in elderly patients undergoing early cholecystectomy [22]. While CCI is a well‐established tool for estimating long‐term prognosis [23, 24], it primarily reflects the cumulative burden of chronic disease and may be less suitable for assessing short‐term surgical risk in acute settings. In contrast, ASA‐PS incorporates dynamic assessments of systemic function and better captures acute physiological derangements [8, 25]. The additional identification of shock status as an independent risk factor further supports this interpretation, as shock represents acute circulatory failure that cannot be adequately reflected by comorbidity‐based indices. Consistent with this concept, a nationwide multicenter study on acute acalculous cholecystitis reported that even Grade III patients experienced acceptable outcomes when upfront cholecystectomy was performed under stable physiological conditions, suggesting that disease severity alone should not preclude operative management [26]. Taken together, our findings suggest that risk stratification in AC should prioritize physiological stability over age or comorbidity burden alone.

Given these considerations, operative strategy and intraoperative decision‐making are critical determinants of acceptable outcomes in physiologically high‐risk patients. The following principles are described to provide context for the institutional surgical approach adopted in this study, rather than as formal technical recommendations. At our institution, a safety‐first strategy underlies the surgical management of AC. Laparoscopic cholecystectomy is the initial approach whenever feasible, even in high‐risk patients. This is consistent with previous reports demonstrating the benefits of minimally invasive surgery in AC [27]. Conversion to open surgery is performed without hesitation in cases of uncontrolled bleeding or uncertain anatomy. To prevent bile duct injury, the critical view of safety technique [7, 28] is routinely attempted, however, it is not pursued when inflammation precludes secure identification. In such circumstances, early bail‐out strategy, most commonly subtotal cholecystectomy is adopted, rather than continuing high‐risk dissection. Accordingly, these operative principles may have contributed to the acceptable perioperative outcomes observed in this cohort, particularly among the TG18 non‐recommended patients.

From a guideline perspective, our results suggest that the current definition of “high‐risk” AC may benefit from refinement. Rather than relying heavily on age‐based or comorbidity‐based criteria, future treatment algorithms should incorporate markers of physiological stability, such as circulatory status and functional reserve, to better identify patients who may safely benefit from emergency surgery despite being classified as “non‐recommended”. Age may be more relevant to outcome assessment, including postoperative recovery and functional independence, than perioperative risk stratification. A large retrospective study evaluating cholecystectomy outcomes by age demonstrated that elderly patients undergoing urgent surgery experienced not only higher postoperative morbidity and mortality but also prolonged hospitalization and increased medical costs, highlighting the substantial impact of surgery on postoperative recovery [29]. Therefore, traditional complication‐based endpoints may not fully capture the clinical burden of emergency cholecystectomy in physiologically vulnerable patients. Hence, future prospective studies are warranted to evaluate outcomes focused on functional recovery and return to preoperative status, which may better reflect patient‐centered benefits and risks in this population.

This study has several limitations. First, this was a retrospective, single‐center study and may therefore be subject to inherent biases. Second, the study was designed as a surgical cohort analysis and included only patients who underwent emergency cholecystectomy. Patients managed with non‐operative strategies (e.g., PTGBD or conservative therapy alone) were not systematically captured. Consequently, selection bias is unavoidable, and our findings reflect outcomes only among surgically treated patients. These results should not be interpreted as evidence that emergency cholecystectomy is broadly feasible for all TG18 non‐recommended patients. Third, although we expanded reporting of clinically meaningful safety endpoints such as bile duct injury, major vascular injury, transfusion‐requiring bleeding, and reoperation, these events were infrequent in our cohort. Therefore, statistical comparisons between groups for these rare events were underpowered and should be interpreted with caution. Future prospective studies including both operative and non‐operative cohorts are warranted to more comprehensively evaluate treatment selection and outcomes in this population.

## 5 Conclusions

Emergency cholecystectomy is not uniformly contraindicated in patients classified as TG18 non‐recommended. A subset of these patients can safely undergo surgery when severe physiological instability is absent. Our findings indicate that perioperative risk in AC is more accurately defined by dynamic physiological parameters, such as ASA‐PS and shock status, rather than by chronological age or comorbidity burden alone. These results support a more individualized approach to surgical decision‐making and suggest that current guideline‐based definitions of high‐risk AC may benefit from further refinement to better reflect real‐world clinical practice.

## Author Contributions

**Satoshi Mii:** conceptualization, methodology, software, data curation, investigation, validation, formal analysis, visualization, project administration, resources, writing – original draft, writing – review and editing. **So Yamaki:** conceptualization, data curation, formal analysis, methodology, validation, investigation. **Daisuke Hashimoto:** conceptualization, data curation, formal analysis, investigation, methodology, project administration, resources, validation. **Kazuki Matsumura:** data curation, formal analysis, investigation, methodology. **Hiroyuki Ishida:** data curation, formal analysis, investigation, methodology. **Hidetaka Miyazaki:** data curation, formal analysis, investigation, methodology. **Yuki Matsui:** data curation, formal analysis, investigation. **Yuji Ikeda:** data curation, formal analysis, investigation. **Tsukasa Ikeura:** data curation, methodology, investigation, validation. **Sohei Satoi:** conceptualization, data curation, formal analysis, investigation, methodology, software, validation, supervision, visualization, project administration, resources, writing – review and editing.

## Funding

The authors have nothing to report.

## Ethics Statement

Approval of the research protocol by an Institutional Reviewer Board: This study was reviewed and approved (Approval Number: 2020131) by the Institutional Review Board of Kansai Medical University, Japan, and complied with STROBE guidelines [30]. All the procedures in this study were performed in accordance with the guidelines of Declaration of Helsinki.

## Conflicts of Interest

S.S. received research funding from Nihon Servier, Amino‐Up Co., Koukamei HSM CO.LTD and Boston Scientific. S.S. serves as an associate editorial board member for the Annals of Gastroenterological Surgery.

## Supporting information


**Table S1:** Overlap of TG18 non‐recommended criteria. (A) Comorbidity‐based criteria used to define TG18 non‐recommended status.
**Table S2:** Classifications of bailout procedures (*n* = 64).
**Table S3:** Detailed breakdown of Clavien–Dindo grade≥III postoperative complications.
**Table S4:** Exploratory risk stratification within the TG18 non‐recommended cohort according to ASA‐PS and shock status.
