# Supplementary material for: Emergency Cholecystectomy in Patients Classified as High Risk According to the Tokyo Guidelines 2018: A Real‐World Analysis
Source: Ann Gastroenterol Surg. 2026 Mar 2;10(4):1298–308. doi: 10.1002/ags3.70209 (PMC13326822; doi:10.1002/ags3.70209)
Supplement: Supplementary file 1 — Table S1: Overlap of TG18 non‐recommended criteria. (A) Comorbidity‐based criteria used to define TG18 non‐recommended status. Table S2: Classifications of bailout procedures (n = 64). Table S3: Detailed breakdown of Clavien–Dindo grade≥III postoperative complications. Table S4: Exploratory risk stratification within the TG18 non‐recommended cohort according to ASA‐PS and shock status. [file AGS3-10-1298-s001.docx]

**Supplementary Table 1: Overlap of TG18 non-recommended criteria**(A) Comorbidity-based criteria used to define TG18 non-recommended status

| Criterion | n (%) |
| --- | --- |
| ASA-PS ≥3 | 80 (31.7) |
| CCI ≥6 | 132 (52.4) |
| Both (ASA-PS ≥3 and CCI ≥6) | 62 (24.6) |

(B) Disease severity (Grade III) and relationship to T18 recommendation status

| Severity category | Total | TG18 non-recommended | TG18 recommended |
| --- | --- | --- | --- |
| Grade III acute cholecystitis | 57 | 49 | 8 |

Footnote:
TG18 non-recommended status was operationally defined based on comorbidity burden (ASA-PS ≥3 and/or CCI ≥6). Grade III severity alone was not used as a defining criterion because TG18 allows early surgery in selected patients with reversible organ dysfunction. Therefore, Grade III was analyzed separately as a severity descriptor.

**Supplementary Table 2: Classifications of bailout procedures (n=64)**

| Open conversion | 35 (54.7) |
| --- | --- |
| Subtotal cholecystectomy with partial wall preservation and cystic duct control | 11 (17.2) |
| Closure-type subtotal resection using ligation or stapling | 16 (25.0) |
| Omental plugging of the gallbladder stump | 1 (1.6) |
| Coverage of an open gallbladder stump using omentum or round ligament of liver | 1 (1.6) |

**Footnote**:
Open conversion was defined as conversion from laparoscopic to open cholecystectomy performed intraoperatively when safe continuation of laparoscopic dissection was judged to be difficult or unsafe due to severe inflammation, fibrosis, or unclear anatomy. In accordance with the TG18 safety framework, open conversion was categorized as a bailout strategy rather than merely a change in surgical approach.
Subtotal cholecystectomy with partial wall preservation and cystic duct control was defined as a procedure in which a portion of the gallbladder wall at the hepatic bed was intentionally preserved to avoid bleeding from the liver parenchyma and injury to peripheral Glissonian pedicles. The cystic duct was controlled by clipping or ligation.
Closure-type subtotal resection using ligation or stapling was defined as a procedure in which the gallbladder was resected in a subtotal fashion and the gallbladder stump was closed by ligation, suturing, or stapling.
Omental plugging of the gallbladder stump was defined as a procedure in which subtotal cholecystectomy was performed and a free omental flap was plugged into the gallbladder stump in cases where secure stump closure was technically difficult.
Coverage of an open gallbladder stump using the omentum or the round ligament of the liver was defined as a procedure in which subtotal cholecystectomy was performed and, in the absence of bile leakage from the stump, the stump was left open and covered with either the omentum or the round ligament of the liver.
For classification purposes, when multiple bailout techniques were used in a single case, cases were categorized according to the primary operative strategy. Accordingly, adjunctive stump-management procedures performed after open conversion (e.g., omental plugging or stump coverage) were not classified as independent bailout categories but were grouped under open conversion.

**Supplementary Table 3: Detailed breakdown of Clavien–Dindo grade ≥III postoperative complications**

| Category | Specific complication | Intervention | Clavien–Dindo grade | n |
| --- | --- | --- | --- | --- |
| Surgical (procedure-related) | Surgical site infection | Wound opening and irrigation | IIIa | 1 |
|  | Bile leakage | ERCP stenting or drain exchange | IIIa | 2 |
|  | Intra-abdominal abscess | Image-guided drainage | IIIa | 1 |
|  | Postoperative bleeding/DIC | Open abdominal management or relaparotomy | IIIb | 2 |
|  | Exploratory laparotomy for suspected perforation | Exploratory laparotomy | IIIb | 1 |
|  | Retained/recurrent CBD stones or cholangitis | ERCP stone extraction or ENBD/ERBD | IIIa | 7 |
|  | ENBD-related intraperitoneal perforation | Emergency laparotomy | IIIb | 1 |
|  | Drain-site herniation of omentum | Wound closure | IIIa | 1 |
| Medical (patient condition-related) | Septic shock with respiratory failure | ICU management with mechanical ventilation | IVa | 1 |
|  | Heart failure | CCU admission | IV | 1 |
|  | Ascites retention | Paracentesis | IIIa | 1 |
|  | Gastrointestinal bleeding | Colonoscopy | IIIa | 1 |
|  | Neurogenic bladder | Catheterization | IIIa | 2 |
|  | Delayed oral intake/gastric dysfunction | Upper endoscopy | IIIa | 1 |
|  | Hyperbilirubinemia/biliary sludge | ERC/ENBD placement | IIIa | 1 |
| Death | Sepsis-related or multiorgan failure |  | V | 4 |
| Total |  |  |  | 30 |

Footnote: Complications were graded according to the Clavien–Dindo classification. Surgical complications were defined as events directly related to the operative procedure, whereas medical complications were defined as systemic or patient-related events. ERCP, endoscopic retrograde cholangiopancreatography; ENBD, endoscopic nasobiliary drainage; CCU, coronary care unit.

**Supplementary Table 4. Exploratory risk stratification within the TG18 non-recommended cohort according to ASA-PS and shock status**

| Subgroup | n | Clavien–Dindo grade ≥III, n (%) | 90-day mortality, n (%) |
| --- | --- | --- | --- |
| No ASA-PS ≥3 and no shock | 70 | 6 (8.6) | 1 (1.4) |
| ASA-PS ≥3 only | 71 | 15 (21.1) | 3 (4.3) |
| Shock (± ASA-PS ≥3) | 9 | 6 (66.7) | 0 (0.0) |
| Total (TG18 non-recommended) | 150 | 27 (18.0) | 4 (2.7) |

Footnote: Patients were stratified according to preoperative ASA-PS and shock status. Shock was defined as the requirement for dopamine ≥5 μg/kg/min and/or norepinephrine. This subgroup analysis was exploratory and hypothesis-generating.
